# Disturbance Is an Important Driver of Clonal Richness in Tropical Seagrasses

**DOI:** 10.3389/fpls.2017.02026

**Published:** 2017-12-05

**Authors:** Kathryn M. McMahon, Richard D. Evans, Kor-jent van Dijk, Udhi Hernawan, Gary A. Kendrick, Paul S. Lavery, Ryan Lowe, Marji Puotinen, Michelle Waycott

**Affiliations:** ^1^School of Science and Centre for Marine Ecosystems Research, Edith Cowan University, Joondalup, WA, Australia; ^2^Western Australian Marine Science Institution, Crawley, WA, Australia; ^3^Marine Science Program, Science and Conservation Division, Department of Biodiversity, Conservation and Attractions, Kensington, WA, Australia; ^4^School of Biological Sciences, University of Western Australia, Crawley, WA, Australia; ^5^UWA Oceans Institute, Crawley, WA, Australia; ^6^School of Biological Sciences, University of Adelaide, Adelaide, SA, Australia; ^7^Pusat Penelitian Oseanografi - Lembaga Ilmu Pengetahuan Indonesia, Jakarta, Indonesia; ^8^Centro de Estudios Avanzados de Blanes, Consejo Superior de Investigaciones Cientificas, Blanes, Spain; ^9^School of Earth Sciences, University of Western Australia, Crawley, WA, Australia; ^10^Indian Ocean Marine Research Centre, Australian Institute of Marine Science, University of Western Australia, Crawley, WA, Australia; ^11^State Herbarium of South Australia, Department of Environment, Water and Natural Resources, Adelaide, SA, Australia

**Keywords:** clonality, disturbance, cyclone, dugong grazing, sea surface temperature (SST)

## Abstract

Clonality is common in many aquatic plant species, including seagrasses, where populations are maintained through a combination of asexual and sexual reproduction. One common measure used to describe the clonal structure of populations is clonal richness. Clonal richness is strongly dependent on the biological characteristics of the species, and how these interact with the environment but can also reflect evolutionary scale processes especially at the edge of species ranges. However, little is known about the spatial patterns and drivers of clonal richness in tropical seagrasses. This study assessed the spatial patterns of clonal richness in meadows of three tropical seagrass species, *Thalassia hemprichii, Halodule uninervis*, and *Halophila ovalis*, spanning a range of life-history strategies and spatial scales (2.5–4,711 km) in Indonesia and NW Australia. We further investigated the drivers of clonal richness using general additive mixed models for two of the species, *H. uninervis* and *H. ovalis*, over 8° latitude. No significant patterns were observed in clonal richness with latitude, yet disturbance combined with sea surface temperature strongly predicted spatial patterns of clonal richness. Sites with a high probability of cyclone disturbance had low clonal richness, whereas an intermediate probability of cyclone disturbance and the presence of dugong grazing combined with higher sea surface temperatures resulted in higher levels of clonal richness. We propose potential mechanisms for these patterns related to the recruitment and mortality rates of individuals as well as reproductive effort. Under a changing climate, increased severity of tropical cyclones and the decline in populations of mega-grazers have the potential to reduce clonal richness leading to less genetically diverse populations.

## Introduction

Clonal plants are represented in the majority of plant lineages (van Groenendael et al., 1996). Due to their ability to reproduce vegetatively, clonal plants have the potential to generate independent offspring through the addition of ramets (shoot, rhizome, and root unit). Clonality is most common in monocots, hence clonal species are common in aquatic habitats where monocots from the Alismatales are prevalent. They also dominate under stressful conditions, such as cold and nutrient-poor environments (van Groenendael et al., 1996; Ye et al., 2016). Populations of clonal plants are comprised of a collection of ramets from a number of genets, where a genet is a single individual (Harper, 1977). The number of genets in a population influences the level of clonality and the number of ramets linked to each genet and the relatedness of these genets affects clonal structure such as the clonal richness, heterogeneity or evenness (Arnaud-Haond et al., 2007). One of the most commonly used measures of clonality is “clonal richness”(R), defined as the number of genotypes (G) relative to the number (N) of samples assessed (R = (G – 1)/(N – 1) (Dorken and Eckert, 2001).

Disturbances, varying both spatially and temporally, can have a profound influence on the clonal structure of a population (Eriksson, 1993). Clonal richness is strongly dependent on the biology of the species, particularly the rate of new genet recruitment (e.g., Zipperle et al., 2010), the mortality rate of existing genets, the competitive interactions between the genets, and how these characteristics interact with the environment (Sebens and Thorne, 1985). For example, intermediate levels of disturbance can facilitate high clonal richness, as it provides opportunities for recruitment of new genets into the population (Sebens and Thorne, 1985). In contrast, high levels of disturbance can lead to low clonal richness due to mass mortality of genets combined with low recruitment of new genets (Sebens and Thorne, 1985; Eriksson, 1993). The latter has been demonstrated using the example of grazing by birds on water plants, with heavy grazing leading to low clonal richness (Hangelbroek et al., 2002). However, enhanced clonal richness under moderate levels of grazing or disturbance has not been demonstrated (Reusch, 2006; Hidding et al., 2014). Under low levels of disturbance, a range of scenarios are possible: with low recruitment and/or the presence of competitively superior genets, low clonal richness will persist (e.g., van Dijk and van Tussenbroek, 2010); but with regular recruitment, even at low rates, clonal richness can be high (e.g., van Tussenbroek et al., 2016).

The clonal richness of plant populations is predicted to decrease with latitude, as higher latitudes are considered more stressful and genets within the population will rely more on clonal reproduction, leading to a lower clonal richness (Stenström et al., 2001; Ye et al., 2014). This is based on evolutionary drivers related to the latitudinal diversity gradient, where species richness and genetic diversity of a range of plant and animal species decline from low to high latitudes (Tittensor et al., 2010; Vellend et al., 2014; Pope et al., 2015; Hernawan et al., 2017). Spatial patterns of within-population clonal richness show contrasting results: declines in clonal richness with latitude (e.g., Stenström et al., 2001); or no relationship (e.g., Olsen et al., 2004; Vik et al., 2010). This highlights the importance of local-scale processes that affect population genetic structure, such as reproduction, dispersal, and bottlenecks, that can strongly influence clonal richness (Olsen et al., 2004). For example, physical isolation and dispersal limitation, the location of the population in relation to the edge of maximum thermal distributional limits and stressful conditions have all been associated with a lower clonal richness (Reusch et al., 1999; Billingham et al., 2003; Olsen et al., 2004; Bienau et al., 2016).

Seagrasses are a polyphyletic group of clonal, marine flowering plants. Like many aquatic plants, they have broad distributions and a low number of species (den Hartog, 1970; Les et al., 1997; Jannsen and Bremer, 2004; Short et al., 2011). In addition, they have a range of life history strategies characterized by differences in the frequency and investment in sexual reproduction, longevity of genets and response to disturbance (Kilminster et al., 2015). These features present a model group to assess broad-scale patterns in clonal richness and the drivers of clonal richness. Historically and in general, seagrasses were considered to rely primarily on clonal reproduction for population persistence; however, over the last two decades population genetic studies have highlighted the high clonal richness of most populations and species (Kendrick et al., 2012, 2017). Positive effects of high clonal richness have been clearly demonstrated, such as a greater resistance to disturbance (Hughes and Stachowicz, 2004, 2009; Reusch et al., 2005; Ehlers et al., 2008; Arnaud-Haond et al., 2010) or by providing greater diversity in plant traits (Evans et al., 2016), especially in responses to perturbations (Salo et al., 2015). Low richness has been observed and attributed to historical conditions over evolutionary timescales such as at the edge of the range in some species (Reusch et al., 1999; Olsen et al., 2004; Evans et al., 2014), the edge of thermal limits to seagrass growth (Billingham et al., 2003), and stressful environmental conditions (Olsen et al., 2004; Diaz-Almela et al., 2007).

The aim of this study is to assess the patterns and drivers of clonal richness in three tropical seagrass species across a range of life-history strategies and spatial scales in Indonesia and NW Australia. This region is a global hotspot of biodiversity for both terrestrial and marine life (Myers et al., 2000; Renema et al., 2008), where the intra-species genetic diversity of marine life tends to decline with latitude (Pope et al., 2015; Hernawan et al., 2017). The region is exposed to a number of disturbances that could affect seagrass such as cyclonic seas and dugong grazing, and is exposed to a range of environmental conditions that could pose a stress to seagrasses such as variation in turbidity and the latitudinal gradient in temperature. Two main questions will be addressed: Does intra-species clonal richness decline with latitude? and What environmental variables best explain spatial patterns of clonal richness?

## Methods

### General approach

Two spatial scales were assessed in this study: a broad spatial scale over 26° latitude from Indonesia to Australia and a fine scale over 8° latitude in the Eastern Indian Ocean, along the NW Australian coast between Broome and Shark Bay. The species *Thalassia hemprichii* was collected over the broader scale. It is a persistent species that is, compared to other tropical seagrass species, relatively slow-growing and resistant to disturbance (Kilminster et al., 2015). It produces buoyant fruits containing a single, direct developing seed, regularly reproduces annually (van Tussenbroek et al., 2006) and is potentially long-lived. Two species, *Halophila ovalis* and *Halodule uninervis* were collected over the finer scale. The life-history strategy of these two species is colonizing, and compared to other tropical species they are fast-growing, not very resistant to disturbance but have an ability to recover rapidly (Kilminster et al., 2015). They both reproduce regularly, developing non-buoyant, dormant seeds that form a seed bank. *H. ovalis* is known to reproduce continuously in some locations with 7–15 seeds per fruit (Waycott et al., 2004) and *H. uninervis* forms one seed per fruit. *H. uninervis* populations sometimes follow an opportunistic life-history strategy, which is intermediate between a colonizing and persistent strategy, and thus this species is more resistant to disturbance and invests less in sexual reproduction compared to colonizing forms (Kilminster et al., 2015). This form is more common in populations with larger morphology (Waycott et al., 2004).

#### Broader-scale study

**Figure 1:**
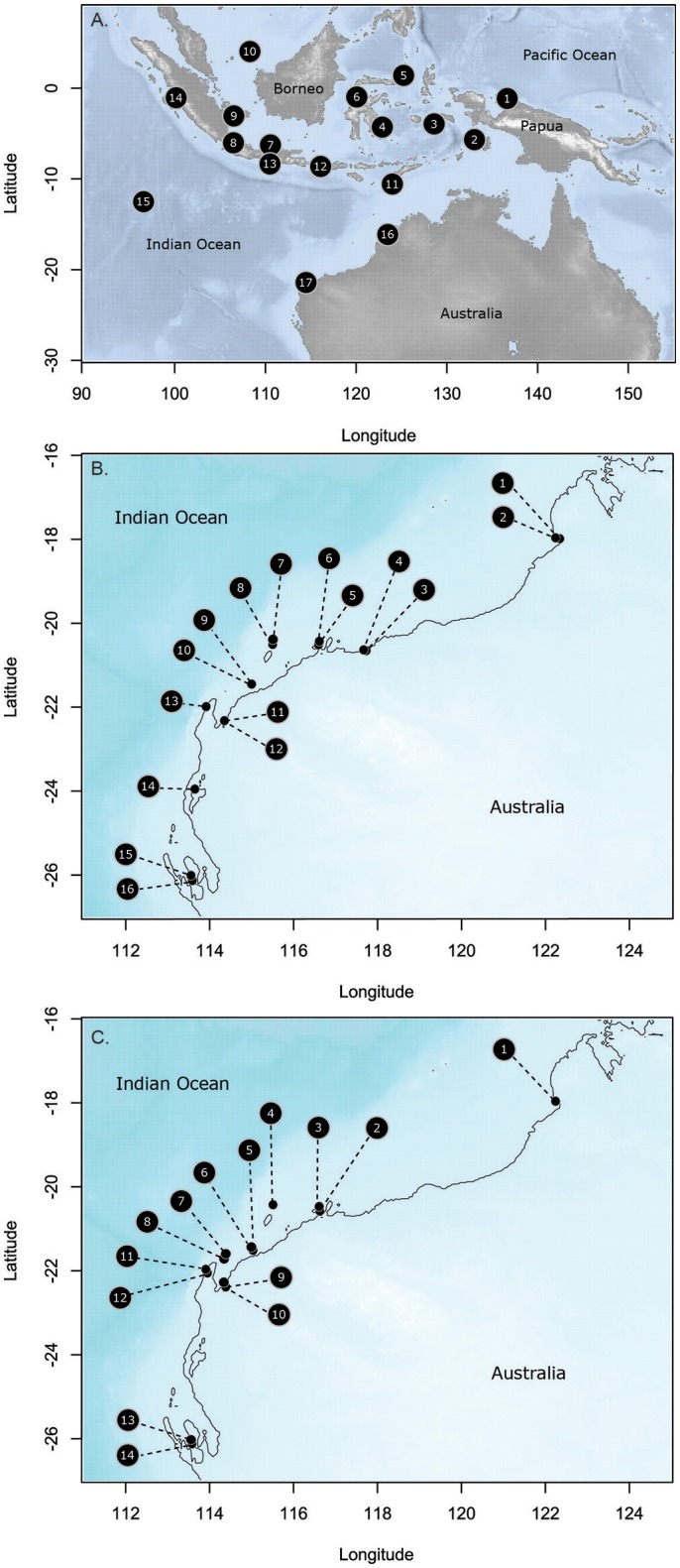
Collection sites for the three species in this study **(A)**. *Thalassia hemprichii*; **(B)**. *Halodule uninervis*; and **(C)**. *Halophila ovalis*. Site names and codes detailed in Supplementary Table 1.

Seventeen sites were sampled throughout Indonesia and on the Indian Ocean side of Australia over 26.6° of latitude (Figure 1, Supplementary Table 1). Distances between sites ranged from 336 to 4 711 km. Populations in the middle of Indonesia (4,6,11,12) represent the center of the distributional range for *T. hemprichii*; whereas, the most southerly Australian sites represent the southern limit of its range. Hernawan et al. (2017) have specifically reported on the genetic structure and connectivity of these sites, and highlighted that the central Indonesian sites have the highest genetic diversity and were the source for dispersal of populations following the last Pleistocene sea level lows ~15,000 years ago when the emerged lands become submerged with sea level rise (Hanebuth et al., 2000). *T. hemprichii* is a common meadow forming species from the equator to Broome (18°S) in Western Australia; south of that, it is not very common.

#### Finer-scale study

Eighteen sites were sampled on the NW coast of Australia in the Eastern Indian Ocean (Figure 1, Supplementary Table 1) over 8.1° of latitude. These were grouped into nine locations, with two sites in each location separated by 2.5–10 km. Sites were selected to maximize the chance that both species were present, although this was not always possible. Fourteen sites had *H. ovalis*, 16 sites had *H. uninervis* and of these 11 sites had both. Both of these species are broadly distributed through the Indo-Pacific, with *H. uninervis* predominantly found in tropical regions, while *H. ovalis* extends into southern temperate waters to 34° of latitude on the Indian Ocean side of Australia (Short et al., 2007). The higher latitude sites were close to the edge of the distribution for *H. uninervis* along the western Australian coast, ~500 km from the range edge at 30.3° of latitude (Waycott et al., 2014). However, further south of the most southerly sites in this study, *H. uninervis* is rare.

### Sample collection

A site was defined as a circular area of 50 m diameter. Fifty samples were collected following two approaches. Where possible, they were collected from random compass bearings and distances along the bearing, which provides a robust method for measuring clonal richness (Arnaud-Haond et al., 2007). Each sample was separated by a minimum of 2 m, and if no seagrass was present at the randomly allocated position, it was collected from the next closest patch of seagrass. If the seagrass was distributed in such a way that this sample design was not possible, then samples were collected haphazardly with a minimum separation of 2 m between samples within a similar area.

Each sample consisted of a seagrass ramet with 1–3 connected shoots. Samples were stored in seawater at ambient temperature until processing. For *H. ovalis* apical meristems and young leaves were collected from each sample, and for *T. hemprichii* and *H. uninervis* the young part of the leaves without epiphytes were collected. All resulting samples were cleaned and stored in silica gel to preserve the DNA within 8 h of collection. A herbarium voucher specimen of each species from each site was also created.

### DNA extraction and genotyping

DNA was extracted from 2 to 3 leaf pairs, growing tips and/or shoots of silica-dried plant material. All extractions were performed using the AGRF extraction service (www.agrf.org.au). Forty six to forty eight samples from each site were genotyped. The lower numbers at some sites were due to running of duplicate samples to improve confidence in the genotpying results.

#### T. hemprichii

Genotyping was conducted on 16 microsatellite markers developed by van Dijk et al. (2014) and Wainwright et al. (2013): Thh5-5 alleles, Thh34-4 alleles, Thh15-6 alleles, TH66-3 alleles, TH37-7 alleles, TH73-5 alleles, TH43-6 alleles, Thh8-5 alleles, TH34-8alleles, Thh41-4 alleles, TH52-9 alleles, TH07-4 alleles, Thh29-4 alleles, Thh1-4 alleles, Thh36-4 alleles, and Thh3-3 alleles. Fluorescently labeled primers were amplified in multiplex reactions using QIAGEN Type-it microsatellite PCR Kit and 0.1–1 ng of DNA template following manufacturer's guidelines. Fragment analysis by capillary separation was performed at the GGF (Georgia Genomic Facility, USA, http://dna.uga.edu) with GGF's size standard 500 ROX. Microsatellite alleles were scored with the Microsatellite plugin in Geneious R7 version 7.1.7 (Biomatters, Auckland, New Zealand).

#### H. uninervis

For *H. uninervis* an alternative genotyping method was developed. A reduced-representation library was generated using a ddRAD approach (double digest Restriction-site Associated DNA) on a small selection of *Halodule* spp. samples from populations in this study area and other regions in northern and eastern Australia. The samples selected for the SNP selection came from a wide range of locations to capture as much genetic diversity as possible. The ddRAD approach roughly follows Peterson et al. (2012) but with some adjustments as described in Villacorta-Rath et al. (2016). The same restriction enzyme combinations were used. For this approach ~200 ng of DNA were digested with restriction enzymes EcoRI (rare cutter) and MseI (frequent cutter). EcoRI and MseI fusion adapters were ligated and libraries amplified with fusion primers containing the Illumina® sequencing adapters by real time PCR on a Roche LightCycler® 96 Instrument. The libraries were size selected by gel electrophoresis on a Pippin Prep (Sage science) and sequenced on a MiSeq (Illumina®) with the MiSeq Reagent Kits v3 and a 2 × 300 bp output. Reads were de-multiplexed, trimmed, and assembled to generate a “Provisional Reference Genome” (PRG, Hird et al., 2011) with both the CLC-Genomic Workbench (Qiagen) and Geneious R7 (Biomatters®) programs. The individual sample reads were then mapped separately to the PRG and loci with high sample coverage and variable SNPs were selected. All selected loci were checked against the NCBI nucleotide databases using the Basic Local Alignment Search Tool (BLAST) function to eliminate loci that might be of bacterial or fungal origin. Any loci with significant similarity to plastid sequences were also eliminated. Where the base calling was consistent with a diploid genome (heterozygote and homozygote sample calls) the locus was considered, 325 passed filter for MassArray® (Agena®) multiplex development. Multiplexes were developed with Assay Design Suite V2.0 following software guidelines and a subset of 135 loci with SNPs were chosen for broadscale genotyping based on primer interactions and flanking region lengths and grouped into four multiplexes of 24–40 loci. Pre-amplification and extension PCRs were performed on all population samples with the iPLEX® Gold genotyping reagent kit and then analyzed on the MassArray® Typer v4.0 analysis software. 80 of the 135 loci successfully amplified consistently across all populations and were used for the data analysis.

#### H. ovalis

Genotyping was conducted using 12 species-specific microsatellite markers developed by Xu et al. (2010) and van Dijk (unpublished), of which 7 (Hpo34-12 alleles, Ho31-2 alleles, Hpo55-12 alleles, Ho20-7 alleles, Ho51-5 alleles, Ho8-7 alleles, Ho5-3 alleles) amplified consistently and were informative. The number of alleles per locus ranged from 2 to 12. Multiplex PCR reactions with fluorescently labeled primers were run, analyzed, and scored as described for *T. hemprichii*.

### Genetic analysis

Each sample site for each species was assessed to test the power of the genetic marker system used to detect unique multilocus genotypes (G). The probability of identity (P_ID_), that two individuals drawn at random within a population will have the same multilocus genotype, was calculated for increasing locus combinations (Waits et al., 2001) using the program GenAlex (v6) (Peakall and Smouse, 2006). Then based on the number of samples collected at the site (N) the expected number of individuals with the same multilocus genotype was estimated. Multilocus genotypes (G's) were identified using the package *poppr* in R (Kamvar et al., 2014). Levels of clonal richness were calculated as R = (G – 1)/(N – 1) (Dorken and Eckert, 2001). Clonal richness as opposed to other measures of clonality such as clonal heterogeneity of evenness was selected as this is the main descriptor of clonality that has been used in the literature to compare against latitudinal patterns or in relation to environmental drivers.

### Species comparisons of clonal richness

To assess if the clonal richness among species differed, a Kruskall Wallis test was used to detect differences between the median and an independent test used to determine if the distribution varied. This was performed in SPSS.

### Latitudinal and spatial patterns of clonal richness

For each species, the correlation of clonal richness at a site with latitude was assessed using the Pearson correlation coefficient (*p* < 0.05). For the finer-scale, where two species were collected at the same sites, the correlation in clonal richness between species was also assessed as described above. This was performed in SPSS.

### Drivers of clonal richness

We focused at the finer-scale with the two species *H. uninervis* and *H. ovalis* as the sampling sites overlapped. *T. hemprichii* over the broader scale was not analyzed as not all environmental factors were available over this scale. We identified a number of key environmental factors that have been demonstrated to influence clonal richness: disturbance (e.g., Eriksson, 1993), environmental stress (e.g., Sebens and Thorne, 1985), isolation and latitude. Two of these were associated with physical disturbance (cyclone disturbance and grazing disturbance), two with environmental stress (temperature and turbidity), and one with geographic isolation, as well as latitude.

#### Cyclone disturbance

High winds during tropical cyclones have the potential to generate extreme waves given sufficient duration and fetch. Consequently, during a cyclone, seagrasses can be exposed to much larger than normal waves that rework bottom sediments, potentially removing individual seagrasses and sometimes entire meadows (e.g., Birch and Birch, 1984; Williams, 1988; K. McMahon personal observations from within the study area, Thevenard Island). We propose that disturbance from cyclonic seas thus has the potential to create a broad-scale physical disturbance. Cyclones frequently cross the north-west Australian coast, and play a major role in shaping ecological communities such as coral reefs (Zinke et al., in press). Over the recent past (1985–2015), the region has been exposed to tropical cyclone activity from 1 to 5 days per year (Puotinen et al., 2016). Due to the potential for a large disturbance, we predict that sites exposed to more frequent and greater cyclone disturbance will have a low clonal richness.

For each site we generated a metric of the annual probability of exposure to cyclonic seas. We reconstructed cyclone generated wind speed every hour along cyclone tracks within the Western Australian region from 1985 to 2015 in a cyclone wind model (McConochie et al., 2004) using data from the International Best Track Archive for Climate Stewardship (IBTrACS—https://www.ncdc.noaa.gov/ibtracs/). To account for the contribution of non-cyclone winds to sea state, at each time step we blended the cyclone generated winds with synoptic winds obtained from the National Center for Atmospheric Research in Boulder, Colorado (CSFR—Climate Forecast System Reanalysis, downloaded from https://climatedataguide.ucar.edu/climate-data/climate-forecast-system-reanalysis-cfsr). We weighted cyclone winds increasingly more heavily with proximity to the cyclone center, and weighted synoptic winds increasingly more heavily with distance beyond three radii of the cyclone eye. Following Puotinen et al. (2016), we used these data to estimate whether large wave conditions (top one-third of wave heights ≥4 m; significant wave height ≥4 m) were possible, assuming adequate fetch. A lack of high resolution bathymetry and reef/island mapping prevented a detailed adjustment for fetch. However, for each cyclone from Nov 2010 to Dec 2015, we used significant wave height, direction and period extracted from the nearest WaveWatch III global hindcast dataset (Tolman, 2009) (downloaded at: http://polar.ncep.noaa.gov/waves/index2.shtml) along with maps of the study sites to assess how exposed the sites likely were to incoming cyclone seas. We then calculated the annual probability that each study site was exposed to such seas for at least 1 h over the time period based on the Poisson distribution using the formula:

(1) $$\text{Pr}(\text{X}\geq \text{1})=1-{\text{e}}^{-\lambda }$$

where λ is the annual average number of times the site was exposed to rough seas each year from 1985 to 2015. This follows prior studies that calculated probabilities of TC landfalls (Tartaglione et al., 2003; Klotzbach, 2011). We assumed that cyclonic seas ≥4 m have the potential to remove seagrass genets, and the more frequently this occurs the more likely that clonal richness would be lower.

#### Dugong grazing disturbance

We propose that grazing by *Dugong dugon* poses an intermediate level of disturbance. A significant proportion of the world's dugong are found in WA, from Shark Bay to the Kimberley, where they feed predominantly on seagrass (Marsh et al., 2012). They generally move through different habitats in small groups to feed (Gales et al., 2004). Dugongs graze by furrowing through the seagrass meadow, removing above and below-ground material and leaving small, bare furrows (Marsh et al., 2012). Therefore, we predict that this scale of disturbance would provide increased opportunities for recruitment from seed, resulting in higher clonal richness.

A number of dugong surveys have been carried out in the region over the last 10 years funded by industry (Chevron and Australia, 2010) and state government (Department of Biodiversity, Conservation and Attractions, unpublished data). However, varying methodologies and the potential for observer bias has prevented a synthesis of these studies that would enable creating a composite density map. These surveys encompassed all of our sites. From them, dugong grazing hotspots were identified based on the areas of highest dugong density (personal communication Amanda Hodgson and Holly Raudino). We used these data to classify each site based on whether a dugong grazing hotspot was present or absent.

#### Temperature and turbidity

Both temperature and light are important physical drivers of seagrass distribution and productivity (Lee et al., 2007; Ralph et al., 2007). As our observations of *H. uninervis* are located close to its temperate range edge, lower temperatures may be more stressful and hence result in lower clonal richness. However, this does not hold for *H. ovalis*, given that the southern limit of our observations are ~10° north of its temperate water boundary. All seagrass species have high light requirements (Ralph et al., 2007), so sites with low light would be predicted to be under more stress and hence have a lower clonal richness.

MODIS (Aqua and Terra) L3 data at 4 km resolution were downloaded from the NASA Goddard Space Flight Center ocean color website (https://oceancolor.gsfc.nasa.gov) for the period 2002–2015 to assess variations in both sea surface temperature (SST) and water column turbidity across the region. Data were extracted at each sample location across a 3 × 3 pixel region that was centered sufficiently offshore (subtidal) to avoid data contamination from any land or shallow reef bathymetry. For SST, data contaminated by clouds or other poor quality data was initially removed using SST quality flags, and data quality was further assessed based on the variance within each 3 × 3 pixel region. A daily SST time series at each location was generated by averaging Aqua and Terra data, and the time series of daily SST were calculated.

Variability in turbidity across the study sites was inferred from MODIS Aqua observations of the diffuse attenuation coefficient at 490 nm (KD_490), a widely used indicator of light attenuation within coastal water columns (Chen et al., 2007; Wang et al., 2009). Data quality was assessed similarly to SST (removing cloudy and other contaminated data) to produce a daily turbidity measure at each location. Based on the depths at the extracted pixel locations (>20 m depth) and relatively turbid conditions of the coastal waters, bottom contamination was estimated to be negligible.

#### Distance from shore

If sites are isolated beyond regular dispersal distances, then if large scale losses occur and no seed-bank is present, the likelihood of recruitment into the disturbed area will be limited, resulting in a greater chance of low clonal richness. Seagrass meadows at our study sites are distributed along the coast and around islands. These islands range from 16 to 75 km from shore and are separated by 70–120 km from the nearest sampled island. However, mapping in this region is limited and there may be not yet discovered seagrass populations within these distances. We used distance from shore, as a proxy for isolation of populations.

#### Statistical analysis: drivers at the finer-scale

The influence of environmental variables on clonal richness was analyzed using generalized additive mixed models (GAMMS; Wood and Scheipl, 2015). GAMMs use a sum of smooth functions to model covariate effects, allowing for more flexible functional dependence of the response variable on the covariates without requiring prior assumptions about the parametric form of the relationship. This was carried out independently for each species, as they were not collected at the same set of sites.

A full-subset method was used to fit models of all possible combinations of variables using the following rules. Prior to analysis several predictor variables were removed due to collinearity >0.8. Latitude was considered but as it was strongly correlated with SST (*r* = 0.96 and 0.98 for *H. uninervis* and *H. ovalis*, respectively) it was not included. As distance from shore correlated with the probability of cyclone disturbance (*r* = 0.86) for *H. uninervis* it was not included in the model for that species. Models were limited to two explanatory variables and models containing variables with correlations exceeding 0.28 were excluded to avoid issues with collinearity among predictor variables, which can cause overfitting and difficulty interpreting results (Graham et al., 2005). The categorical variable of dugong grazing disturbance was included in the model as a factor. All models within two AICc-scores (Aikake Information Criterion corrected for a small sample size) of the best AICc-score were considered, as this is the threshold level for models with reasonable likelihood (Burnham and Anderson, 2003). However, the best model was selected based on parsimony and contained the fewest explanatory variables within two AICc units of the model with the lowest AICc-value (Burnham and Anderson, 2003). Finally, the *r*^2^-values were used to provide an indication of the predictive power of the model (Burnham and Anderson, 2003). The distribution of the response variables fitted a Gaussian distribution. The distribution of predictor variables was visually inspected and appropriate data transformations applied where necessary. Temperature (SST) was log transformed and turbidity (KD_490_) was square root transformed. Distance from shore was square root transformed for *H. ovalis*.

## Results

### Clonal richness determination

All marker sets had a high probability of detecting MLGs generally with *p* < 0.001 but in some cases < 0.05. The expected number of samples with the same MLG was < 1 in all but one case, at a *H. ovalis* site (Supplementary Table 1).

### Species comparisons

**Figure 2:**
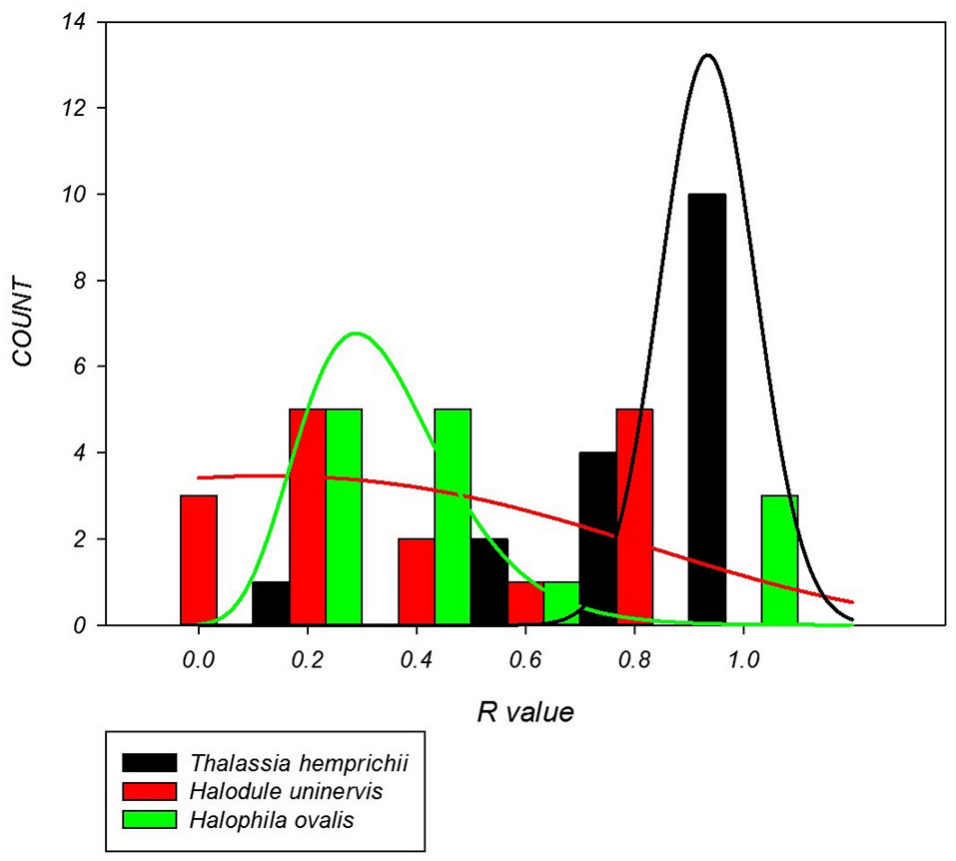
Distribution of clonal richness for each seagrass species based on 14–17 sites per species. *H. uninervis* has the lowest median (*R* = 0.19) and is most skewed to lower clonal richness, *H. ovalis* is similar (*R* = 0.27) and *T. hemprichii* has a much higher median clonal richness (0.74) with a right skewed distribution.

There were significant differences in the clonal richness between species based on the median (*p* = 0.006) and distribution (*p* < 0.001). Colonizing species, *H. ovalis* (*R* = 0.27) and *H. uninervis* (*R* = 0.19) had a lower median clonal richness and a more left skewed distribution than the persistent species, *T. hemprichii* (*R* = 0.74, Figure 2).

### Latitudinal and spatial patterns in clonal richness

There were no significant correlations between clonal richness and latitude for each species (Supplementary Figure 1, Supplementary Table 2). However, there was a significant correlation of clonal richness between species which co-occurred at sites (*R* = 0.803, *p* < 0.01) indicating local scale-processes may be more critical in determining clonal richness (Supplementary Figure 1, Supplementary Table 2).

### Drivers of clonal richness

**Figure 3:**
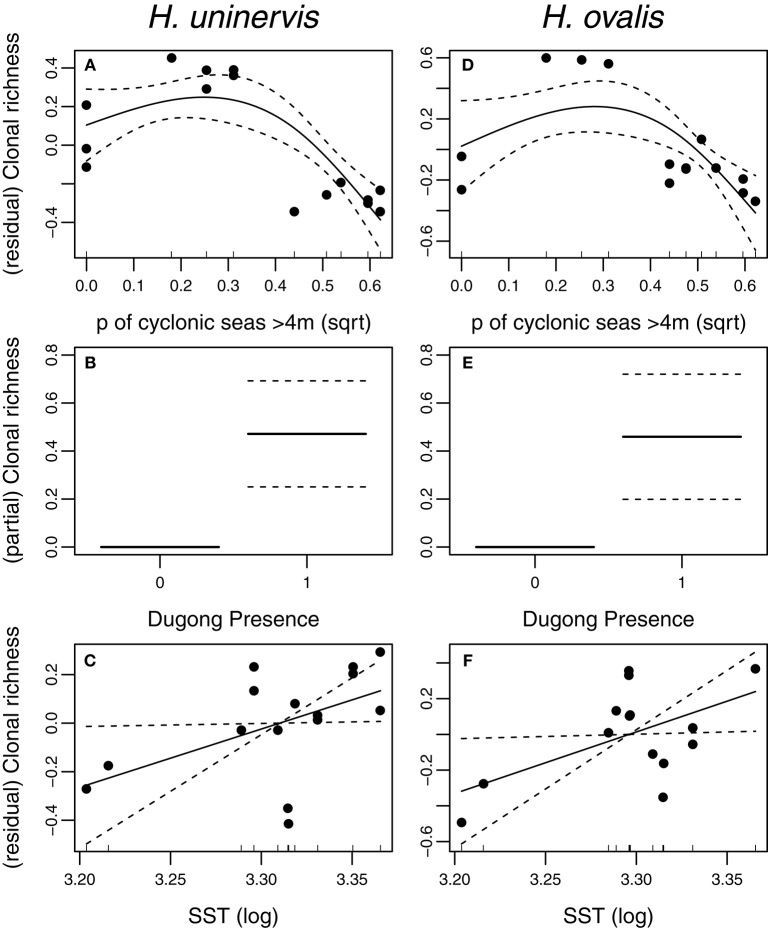
Partial and residual biplots of relationships between predictor variables within two AICc of the top score for *H. uninervis* and *H. ovalis* clonal richness. Models were fitted using GAMMs with the solid black line representing the estimated smoothing curve and the dashed lines representing ± SE of the estimate. The most parsimonious model for *H. uninervis* was **(A)** probability of cyclonic seas. There were two most parsimonious models for *H. ovalis* **(D)** Probability of cyclonic seas, and **(E)** Dugong presence. SST (Sea surface Temperature) **(C,F)** was included in a top model when combined with dugong **(B,E)** for both species.

Two models, exposure to cyclonic wave conditions and SST + dugong grazing presence were well-supported in explaining the clonal richness of *H. uninervis* (Table 1). The most parsimonious was the probability of cyclonic wave conditions (*r*^2^ = 0.68). This model showed the highest clonal richness at intermediate levels of cyclone disturbance, a lower clonal richness at low disturbance levels and even lower at very high probabilities of cyclonic seas (Figure 3). In the other well-supported model, the clonal richness of *H. uninervis* increased with sea surface temperature and in the presence of dugongs (Figure 3, Table 1). The probability of cyclonic seas had the highest relative importance (0.57), followed by dugong grazing (0.37) and SST (0.24) (Table 2).

**Table 1:** Outputs of the generalized additive mixed models (GAMMs) for predicting the clonal richness of *Halodule uninervis* and *Halophila ovalis* in NW Australia.

**Species**	**Models**	**AICc**	**delta.AICc**	**wi.AICc**	***r*^2^**
*Halodule uninervis*	**cyclone.sqrt**	**1.114**	**0**	**0.572**	**0.677**
	SST.log+dugong	3.078	1.964	0.214	0.639
	dugong	4.002	2.888	0.135	0.505
	KD490.sqrt	6.239	5.125	0.044	0.515
	dugong+SST.log.by.dugong	7.714	6.6	0.021	0.64
	null	11.378	10.264	0.003	0
	SST.log	12.51	11.396	0.002	0.273
*Halophila ovalis*	SST.log+dugong	8.525	0	0.315	0.566
	**cyclone.sqrt**	**9.164**	**0.638**	**0.229**	**0.533**
	**dugong**	**9.425**	**0.9**	**0.201**	**0.382**
	KD490.sqrt	10.566	2.041	0.113	0.33
	dugong+SST.log.by.dugong	11.59	3.065	0.068	0.624
	null	12.863	4.338	0.036	0
	Dist.shore.sqrt+KD490.sqrt	14.399	5.874	0.017	0.34
	SST.log	15.069	6.544	0.012	0.076
	Dist.shore.sqrt	15.505	6.98	0.01	0.066

*The Akaike Information Criterion corrected for small sample sizes (AICc), AIC differences (delta AIC_c_), model weights (wi.AICc), and the coefficient of determination (r2) are also reported. The best supported models are within 2 delta AIC_c_ and the most parsimonious model with best predictive power is in bold. The predictor variables are: Dist shore (the distance of each site from shore); Cyclone (the probability of exposure to cyclonic seas > 4m); KD490 (a remotely sensed proxy of water column turbidity); SST (Sea surface temperature); and Dugong (presence of a dugong grazing hotspot). The transformation used for each predictor variable is also included*.

**Table 2:** Variable importance of the predictor variables from the generalized additive mixed models (GAMMs) for predicting the clonal richness of *Halodule uninervis* and *Halophila ovalis* in North Western Australia.

	**Dist shore**	**Cyclone**	**KD490**	**SST**	**Dugong**
*Halodule uninervis*	NA	0.572	0.044	0.240	0.370
*Halophila ovalis*	0.027	0.229	0.130	0.395	0.584

*See Table 1 legend for description of predictor variables. Colors indicate the relative importance and are scaled from green—least important to red—most important*.

The models that best predicted *H. ovalis* clonal richness were the same as for *H. uninervis*, but also included a third model, dugong grazing as a single variable. The most parsimonious were the two models with a single predictor, cyclone disturbance and dugong grazing hotspot (Table 1). *H. ovalis* clonal richness was highest with intermediate levels of cyclone disturbance and lowest at both a low and high probability of cyclonic seas (Figure 3, Table 1). Clonal richness also increased in the presence of dugongs and with temperature (Figure 3, Table 1). For *H. ovalis*, the relative importance of the predictor variables was greatest for dugong grazing (0.58), followed by sea surface temperature (0.39), then cyclone disturbance (0.23) (Table 2). SST by itself is a very poor predictor of clonal richness in both species. It ranked below the null model based on AICc selection but added some value to dugong grazing as it was ranked second in the variable importance table for both species (Table 2).

## Discussion

### Disturbance and SST predict clonal richness

Disturbance combined with sea surface temperature strongly explained spatial patterns in clonal richness in the two colonizing species assessed. But there were no significant patterns with latitude for all three species, where two species were at or very close to their range edge. This finding points to the importance of disturbance and environmental conditions in influencing the clonal richness of populations of colonizing species of seagrass, rather than evolutionary timescale processes that have been shown to influence latitudinal genetic diversity patterns in this region (Pope et al., 2015; Hernawan et al., 2017). Studies that have identified latitudinal patterns in clonal richness (e.g., Stenström et al., 2001) have extended into very high latitudes latitudes, quite dissimilar to the more tropical environment of this study. We recommend further studies to investigate these patterns over broader latitudinal scales.

Over ecological time-scales and across multiple generations, the clonal richness of a population is dependent on the recruitment rate of new genets, the longevity or mortality rates of these genets and the interactions between genets (Sebens and Thorne, 1985; Eriksson, 1993). Disturbance from tropical cyclones and dugong grazing, as well as SST can all directly impact these processes, but the nature or level of disturbance appears to be important. The study area is a region of high cyclone activity, with 1–5 days exposure every year and sites with a high probability of cyclone disturbance are likely to experience cyclones in multiple years. Extreme cyclone disturbance can clearly provide a mechanism for increased genet mortality, either completely removing all genets or the majority of the genets. When this is repeated over multiple years, the population could be reduced further leading to low clonal richness. In addition, if the seed bank is removed or there is not enough time between repeated cyclones for the seed bank to develop, this would reduce the potential for new genets to enter the population, limiting clonal richness. As dispersal between meadows is predicted to be quite restricted in these species due to their non-buoyant fruits and seeds (Kendrick et al., 2012), recovery of cyclone impacted sites by dispersal of recruits from adjacent meadows is likely to be limited. This was seen in our study where sites with high probabilities of cyclonic seas >4 m had low clonal richness. But at sites with intermediate levels of disturbance from cyclonic seas, where they are sometimes exposed to cyclones over a year, clonal richness was highest. An intermediate probability of cyclonic seas is still likely to lead to some genet mortality, but with more time between disturbance events, the seed bank can develop, enabling the recruitment of new genets into the population. At low levels of disturbance, the model of Sebens and Thorne (1985) predicts clonal richness could range from low to high, depending on the competitive interactions between genets and the recruitment rate of new genets into the population. We found that meadows with low levels of disturbance indeed had lower clonal richness than meadows exposed to intermediate levels of disturbance; however, the relative amount of clonal richness varied between the two species, potentially driven by recruitment rates of new genets or genet interactions.

Regular-repeated grazing, as long as it does not result in increased mortality rates of genets (e.g., Hangelbroek et al., 2002), could lead to increased recruitment (see Inglis, 2000) through providing microhabitats for seeds to germinate (e.g., Reisch and Scheitler, 2009), seedlings to establish and enhance clonal richness. In this region, dugongs generally feed in small groups (Hodgson et al., 2008), so almost complete removal of meadows is unlikely, although in other densely populated locations (e.g., Moreton Bay, Australia) it has been observed (Preen, 1995). There is a feedback mechanism with dugong grazing, where disturbance, here the fragmentation of the rhizome from grazing, stimulates flowering, fruiting, and hence the density of the seed bank (Alexandre et al., 2005; Cabaço and Santos, 2012), leading to the potential for more recruits. Dugongs can disperse seeds from one population to another (McMahon et al., 2014; Kendrick et al., 2017; Tol et al., 2017), providing another pathway for recruitment and an increase in clonal richness among grazed sites for seagrass species with hard-coated seeds.

Temperature on its own did not explain patterns in clonal richness, identified through both the latitudinal assessment, where latitude can be a proxy for temperature, and the GAMM results. However, in combination with dugong grazing it was an important driver of clonal richness. Temperature may be important for the effect it has on dugong grazing pressure and/or seagrass reproduction. Higher temperatures are known to increase reproductive effort for some seagrass species or the number of reproductive events within a year (de Cock, 1981). For example *H. ovalis* sexually reproduces annually, over the summer period in temperate regions (e.g., Hillman et al., 1995) but can have multiple flowering times in the tropics (Waycott et al., 2007). Therefore, if higher temperatures reflect greater sexual reproductive output over a year, this could manifest in an increase in the number of recruitment times. The effect of temperature on dugong grazing pressure is not known, although lower temperatures limit dugong distribution over its distribution range and at the local scale (Anderson, 1994; Marsh et al., 2012). If higher temperatures result in more grazing at a site, then this combined with increased reproductive output through dugong grazing, could lead to the higher clonal richness observed in this study.

Despite our observations of *H. uninervis* being located closer to its range edge (~500 km) compared to *H. ovalis* (~1,200 km), the clonal richness of both species was explained by the same drivers. This may be due to the similar life-history strategy of the two species; colonizers that are not very resistant to disturbance, potentially have short life-spans and use dormant seed banks as a mechanism for recovery (Ooi et al., 2014; Kilminster et al., 2015). Therefore, the two critical processes influencing clonal richness, recruitment of new individuals into the population and mortality or longevity of genets (Eriksson, 1993) may respond in the same way to disturbance and temperature. For example, in relation to the mortality of genets with cyclone disturbance, both species are quite small, with a similar investment in below-ground material that would act as a poor anchor for plants during cyclonic seas. Recruitment and introduction of new genets could occur via three main mechanisms: settlement of vegetative fragments, germination of the seed bank or dispersal of seeds via dugongs and the subsequent germination of these seeds (McMahon et al., 2014). Recruitment via vegetative fragments has been recorded in both species (Hall et al., 2006; Wu et al., 2016) with similar durations of viability (maximum ~4 weeks) as well as via seed banks (Inglis, 2000; Rasheed, 2004) and through biotic dispersal by dugongs (Tol et al., 2017). They are also both commonly grazed by dugongs (Marsh et al., 2012) so small-scale, repeated grazing has the potential to enhance recruitment. In fact, a higher density of *H. uninervis* seeds has been observed in dugong feeding scars and they have an increased probability of seed germination and recruitment following grazing (Inglis, 2000).

### Species differences

The main difference between the two colonizing species was the absolute levels of clonal richness; *H. uninervis* had a more skewed distribution with lower clonal richness. It could be possible that this is due to the type of markers used (Arnaud-Haond et al., 2007) but as the SNP's marker system employed for *H. uninervis* had a higher probability of detecting MLG's than the microsatellite system used for *H. ovalis*, this is unlikely to be the case. An alternate hypothesis for the lower clonal richness in *H. uninervis* is the relative rates of recruitment of new genets into meadows, where *H. uninervis* has a lower recruitment rate compared to *H. ovalis*. Both species have the potential to produce seed at each node on a ramet, although at peak flowering times, usually <20% of nodes have flowers, but *H. ovalis* has more seeds per fruit, up to 15 as opposed to only one in *H. uninervis*. So a relatively lower seed bank density could reduce the number of recruits entering the population following a large scale disturbance impacting clonal richness in *H. uninervis*. Populations with low reproductive effort have also been recorded for *H. uninervis* (Olesen et al., 2004) indicating that low seed production could also contribute to this process. To understand the conditions of the local populations in this study, further research is required on the reproductive biological processes and recruitment mechanisms.

Compared to the colonizing species, the persistent species, *T. hemprichii* generally had much higher clonal richness. From a life-history perspective, persistent species are characterized by being more resilient to disturbance, having longer life-spans and slower recovery rates, through a combination of recruitment from seed and vegetative expansion (Kilminster et al., 2015). Therefore, based on the “Repeated seedling recruitment” (RSR) strategy described by Eriksson (1993), over time, continual recruitment from seed, even at a low rate, can lead to a high clonal richness, especially with long-lived genets. Moderate to high levels of clonal richness have been observed in many meadows of persistent species (e.g., Arnaud-Haond et al., 2010; van Dijk and van Tussenbroek, 2010; Sinclair et al., 2014). Although factors driving clonal richness for *T. hemprichii* were not investigated in this study due to the challenge of obtaining a similar set of environmental drivers over the broader spatial scale, disturbance can have contrasting effects. For example van Dijk and van Tussenbroek (2010) showed sites with higher disturbance from wave energy had higher clonal richness compared to more protected sites (e.g., van Dijk and van Tussenbroek, 2010) but Diaz-Almela et al. (2007) found meadows exposed to aquaculture pollution had lower clonal richness. Due to the effects of the environment and the interactions with genet mortality and recruitment, it is likely species with different life-history strategies would respond differently to disturbance. As was shown in this study, colonizing species that are not as long-lived and less resistant to disturbance have a more dynamic boom and bust cycle and thus may be more likely to have a lower clonal richness. Clearly more research is required to investigate patterns of clonal richness in relation to the life-history strategy of seagrasses.

A meta-analysis on clonal, terrestrial plants did not find a consistent pattern with life-history strategy and clonal richness (Honnay and Jacquemyn, 2008). However, studies in the coral literature show similar patterns. In colonizing coral species, clonal richness declines with increasing physical disturbance and this has been explained through the re-attachment of fragments post disturbance (Foster et al., 2013), and under extreme high levels of disturbance the majority of genets can be removed from a location, leading to very low clonal richness (Coffroth and Lasker, 1998). Indeed, a recent study in NW Australia in the same area as this study, found that the colonizing coral *Pocillopora damicornis* had lower clonal richness (Thomas et al., 2014, 2017) compared to the stress tolerant and persistent *Cypahstrea micropthalma* (Darling et al., 2012; Burt et al., 2016; Howells et al., 2016) collected at the same locations where all individuals collected had unique genotypes (Evans unpublished data).

### Management implications

This study has highlighted three key drivers of seagrass clonal richness: exposure to cyclonic seas, dugong grazing and average SST, all of which are changing under human pressures. It is predicted that in the absence of large reductions in greenhouse gas emissions the number of cyclones will not change but the severity of the cyclones will increase, with a greater proportion being more severe (Sobel et al., 2016). Based on the findings in this study, such increases will potentially lead to more sites with low clonal richness or local extinctions. Interestingly, the coast of Western Australia has the highest frequency of cyclone crossings in the Indo-Pacific region with an average of three cyclones per year (Lough, 1998). Comparisons of the levels of clonal richness and its drivers would be fruitful in other regions to assess whether these patterns hold where cyclones are not as prevalent.

Globally the dugong population is declining (Marsh and Sobtzick, 2015) related to a range of threats such as incidental by-catch, hunting, boat strikes, and habitat loss, often associated with land-use (Marsh et al., 2012; Quiros et al., 2017). This study has demonstrated that reduced grazing pressure can have negative impacts on clonal richness. A declining dugong population presents a negative feedback resulting in reduced grazing pressure on seagrass, which could lead to reduced seagrass resilience due to a lowered clonal richness and associated loss of within population diversity. Jackson et al. (2001) proposed that collapses of seagrass meadows and other marine habitats could be due to the massive reductions in mega-grazers compared to historical levels, and this study provides one possible mechanism for this.

Unlike the global trajectories for cyclone exposure and dugong grazing pressure, which do not bode well for tropical seagrass, a warming climate may have a positive effect on clonal richness, as long as the temperature does not increase above the normal tolerance limits of the populations. The positive relationship between average temperature and clonal richness of seagrass meadows implies that increased temperature, particularly in the more temperate populations, may stimulate more flowering and seed set, and thus increase local recruitment, clonal richness, and the genetic adaptive capacity. However, while the increasing temperatures in temperate regions may seem like a positive story, the recent reduction of temperate species seagrass meadows in the subtropical part of this study region, associated with heat waves (e.g., Fraser et al., 2014) demonstrate negative impacts where temperature exceeds the tolerance limits for those species and the taxa associated with them (Thomson et al., 2015). For the tropical species in this study, this may mean a range reduction at the lower latitudes and expansion at the higher latitudes (Hyndes et al., 2016).

Clonal richness has significant implications for genetic resilience as low clonal richness implies a small population with low genetic diversity and limited ability to resist and adapt to environmental change (Hughes and Stachowicz, 2004, 2009; Reusch et al., 2005; Ehlers et al., 2008; Engelhardt et al., 2014; Salo et al., 2015). A low clonal richness also means a low potential to produce viable offspring and develop a seed bank, an important process to enable recovery from extreme events that result in mass mortality. Consequently, to effectively manage and conserve seagrass habitats, clonal richness, and the factors affecting it such as dugong density should be understood to help guide decision-making. For example, Engelhardt et al. (2014) identified that clonal richness was very important for increasing the probability of flowering and seed set in the clonal aquatic plant, *Vallisneria*, and to manage threatened habitats, recommended introducing genotypes with an appropriate phenotype set to enhance reproductive potential and resilience. Clonal richness of seagrass meadows in a local and regional context, should be considered as part of the tool box for informing conservation management decisions (Unsworth et al., 2015), and those meadows with low clonal richness should be flagged as at greater risk as they are less resilient to environmental change.

## Author contributions

KM, RE, UH, PL, and GK: designed study; KM, RE, UH, and GK: collected samples; KM, UH, and KvD: carried out genetic laboratory work and analysis; MP: generated cyclone dataset; RL: generated temperature and light dataset; RE: carried out GAMMs analysis; KM and RE: led writing, and all authors edited and reviewed manuscript.

### Conflict of interest statement

The authors declare that the research was conducted in the absence of any commercial or financial relationships that could be construed as a potential conflict of interest.

## References

[B1] Alexandre A. Santos R. Serrao E. (2005). Effect of clam harvesting on sexual reproduction of the seagrass *Zostera noltii*. Mar. Ecol. Prog. Ser. 298, 115–122. 10.3354/meps298115

[B2] Anderson P. K. (1994). Dugong distribution, the seagrass *Halophila spinulosa*, and thermal environment in winter in deeper waters of eastern Shark Bay, Western Australia. Wildl. Res. 21, 381–388. 10.1071/WR9940381

[B3] Arnaud-Haond S. Duarte C. M. Alberto F. Serrão E. A. (2007). Standardizing methods to address clonality in population studies. Mol. Ecol. 16, 5115–5139. 10.1111/j.1365-294X.2007.03535.x 17944846

[B4] Arnaud-Haond S. Marba N. Diaz-Almela E. Serrao E. A. Duarte C. M. (2010). Comparative analysis of stability-genetic diversity in seagrass (*Posidonia oceanica*) meadows yields unexpected results. Estuaries Coasts 33, 878–889. 10.1007/s12237-009-9238-9

[B5] Bienau M. J. Eckstein R. L. Otte A. Durka W. (2016). Clonality increases with snow depth in the arctic dwarf shrub *Empetrum hermaphroditum*. Am. J. Bot. 103, 2105–2114. 10.3732/ajb.1600229 27919923

[B6] Billingham M. Reusch T. B. H. Alberto F. Serrao E. A. (2003). Is asexual reproduction more important at geographical limits? Mar. Ecol. Prog. Ser. 265, 77–83. 10.3354/meps265077

[B7] Birch W. R. Birch M. (1984). Succession and pattern of tropical intertidal seagrasses in Cockle Bay, Queensland, Australia: a decade of observations. Aquat. Bot. 19, 343–367. 10.1016/0304-3770(84)90048-2

[B8] Burnham K. P. Anderson D. R. (2003). Model Selection and Multimodel Inference: a Practical Information-Theoretic Approach. New York, NY: Springer Science & Business Media.

[B9] Burt J. A. Smith E. G. Warren C. Dupont J. (2016). An assessment of Qatar's coral communities in a regional context. Mar. Pollut. Bull. 105, 473–479. 10.1016/j.marpolbul.2015.09.025 26410180

[B10] Cabaço S. Santos R. (2012). Seagrass reproductive effort as an ecological indicator of disturbance. Ecol. Indic. 23, 116–122. 10.1016/j.ecolind.2012.03.022

[B11] Chen Z. Chuanmin H. Muller-Karger F. (2007). Monitoring turbidity in Tampa Bay using MODIS/Aqua 250-m imagery. Remote-sens. Environ. 109, 207–220. 10.1016/j.rse.2006.12.019

[B12] Chevron and Australia (2010). Dugong Research Plan: Wheatstone Project. Perth, WA: Chevron Australia Pty Ltd.

[B13] Coffroth M. A. Lasker H. R. (1998). Population structure of a clonal gorgonian coral: the interplay between clonal reproduction and disturbance. Evol. Ecol. 52, 379–393. 28568334 10.1111/j.1558-5646.1998.tb01639.x

[B14] Darling E. S. Alvarez-Filip L. Oliver T. A. McClanahan T. R. Côté I. M. (2012). Evaluating life-history strategies of reef corals from species traits. Ecol. Lett. 15, 1378–1386. 10.1111/j.1461-0248.2012.01861.x 22938190

[B15] de Cock A. (1981). Influence of temperature and variations in temperature on flowering in *Zostera marina* l. under laboratory conditions. Aquat. Bot. 10, 125–131. 10.1016/0304-3770(81)90015-2

[B16] den Hartog C. (1970). Sea-Grasses of the World. Amsterdam: North Holland Publishing Company.

[B17] Diaz-Almela E. Arnaud-Haond S. Vliet M. S. Alvarez E. Marba N. Duarte C. M. (2007). Feed-backs between genetic structure and perturbation-driven decline in seagrass (*Posidonia oceanica*) meadows. Conserv. Genet. 8, 1377–1391. 10.1007/s10592-007-9288-0

[B18] Dorken M. E. Eckert C. G. (2001). Severely reduced sexual reproduction in northern populations of a clonal plant, *Decodonverticillatus* (Lythraceae). J. Ecol. 89, 339–350. 10.1046/j.1365-2745.2001.00558.x

[B19] Ehlers A. Worm B. Reusch T. B. H. (2008). Importance of genetic diversity in eelgrass *Zostera marina* for its resilience to global warming. Mar. Ecol. Prog. Ser. 355, 1–7. 10.3354/meps07369

[B20] Engelhardt K. A. M. Lloyd M. W. Neel M. C. (2014). Effects of genetic diversity on conservation and restoration potential. at individual, population, and regional scales. Biol. Conserv. 179, 6–16. 10.1016/j.biocon.2014.08.011

[B21] Eriksson O. (1993). Dynamics of genets in clonal plants. Trends Ecol. Evol. 8, 313–316. 10.1016/0169-5347(93)90237-J 21236180

[B22] Evans S. M. Sinclair E. A. Poore A. G. Bain K. F. Vergés A. (2016). Genotypic richness predicts phenotypic variation in an endangered clonal plant. PeerJ 4:e1633. 10.7717/peerj.1633 26925313 PMC4768672

[B23] Evans S. M. Sinclair E. A. Poore A. G. B. Steinberg P. D. Kendrick G. A. Verges A. (2014). Genetic diversity in threatened *Posidonia australis* seagrass meadows. Conserv. Genet. 15, 717–728. 10.1007/s10592-014-0573-4

[B24] Foster N. L. Baums I. B. Sanchez J. A. Paris C. B. Chollett I. Agudelo C. L.. (2013). Hurricane-driven patterns of clonality in an ecosystem engineer: the Caribbean coral *Montastraea annularis*. PLoS ONE 8:e53283. 10.1371/journal.pone.0053283 23308185 PMC3538762

[B25] Fraser M. W. Kendrick G. A. Statton J. Hovey R. K. Zavala-Perez A. Walker D. I. (2014). Extreme climate events lower resilience of foundation seagrass at edge of biogeographical range. J. Ecol. 102, 1528–1536. 10.1111/1365-2745.12300

[B26] Gales N. McCauley R. D. Lanyon J. Holley D. (2004). Change in abundance of dugongs in Shark Bay, Ningaloo and Exmouth Gulf, Western Australia: evidence for large-scale migration. Wildl. Res. 31, 283–290. 10.1071/WR02073

[B27] Graham N. Dulvy N. Jennings S. Polunin N. (2005). Size-spectra as indicators of the effects of fishing on coral reef fish assemblages. Coral Reefs 24, 118–124. 10.1007/s00338-004-0466-y

[B28] Hall L. M. Hansiak D. Virnstein R. W. (2006). Fragments of the seagrasses *Halodule wrightii* and *Halophila johnsonii* as potential recruits in Indian River Lagoon, Florida. Mar. Ecol. Prog. Ser. 310, 109–117. 10.3354/meps310109

[B29] Hanebuth T. Stattegger K. Grootes P. (2000). Rapid flooding of the Sunda shelf: a late-glacial sea-level record. Science 288, 1033–1035. 10.1126/science.288.5468.1033 10807570

[B30] Hangelbroek H. H. Ouborg N. J. Santamaría L. Schwenk K. (2002). Clonal diversity and structure within a population of the pondweed *Potamogeton pectinatus* foraged by Bewick's swans. Mol. Ecol. 11, 2137–2150. 10.1046/j.1365-294X.2002.01598.x 12296955

[B31] Harper J. L. (1977). Population Biology of Plants. London: Academic Press

[B32] Hernawan U. E. van Dijk K.-j. Kendrick G. A. Feng M. Biffin E. Lavery P.. (2017). Historical processes and contemporary ocean currents drive genetic structure in the seagrass *Thalassia hemprichii* in the Indo-Australian Archipelago. Mol. Ecol. 26, 1008–1021. 10.1111/mec.13966 27997066

[B33] Hidding B. Meirmans P. G. Klaassen M. de Boer T. Ouborg N. J. Wagemaker C. A. M. (2014). The effect of herbivores on genotypic diversity in a clonal aquatic plant. Oikos 123, 1112–1120. 10.1111/oik.01136

[B34] Hillman K. McComb A. J. Walker D. I. (1995). The distribution, biomass and primary production of the seagrass *Halophila ovalis* in the Swan-Canning Estuary, Western- Australia. Aquat. Bot. 51, 1–54. 10.1016/0304-3770(95)00466-D

[B35] Hird S. M. Brumfield R. T. Carstens B. C. (2011). RGmatic: an efficient pipeline for collating genome-enriched second-generation sequencing data using a ‘provisional-reference genome.’ Mol. Ecol. Resour. 11, 743–748. 10.1111/j.1755-0998.2011.03005.x 21676202

[B36] Hodgson A. J. Marsh H. Gales N. Holley D. K. Lawler I. (2008). Dugong Population Trends Across Two Decades in Shark Bay, Ningaloo Reef and Exmouth Gulf, Western Australia. Department of Environment and Conservation.

[B37] Honnay O. Jacquemyn H. (2008). A meta-analysis of the relation between mating system, growth form and genotypic diversity in clonal plant species. Evol. Ecol. 22, 299–312. 10.1007/s10682-007-9202-8

[B38] Howells E. J. Ketchum R. N. Bauman A. G. Mustafa Y. Watkins K. D. Burt J. A. (2016). Species-specific trends in the reproductive output of corals across environmental gradients and bleaching histories. Mar. Pollut. Bull. 105, 532–539. 10.1016/j.marpolbul.2015.11.034 26608503

[B39] Hughes A. R. Stachowicz J. J. (2004). Genetic diversity enhances the resistance of a seagrass ecosystem to disturbance. Proc. Natl. Acad. Sci. U.S.A. 101, 8998–9002. 10.1073/pnas.0402642101 15184681 PMC428461

[B40] Hughes A. R. Stachowicz J. J. (2009). Ecological impacts of genotypic diversity in the clonal seagrass *Zostera marina*. Ecology 90, 1412–1419. 10.1890/07-2030.1 19537560

[B41] Hyndes G. A. Heck K. L. Vergés A. Harvey E. S. Kendrick G. A. Lavery P. S.. (2016). Accelerating tropicalization and the transformation of temperate seagrass meadows. Bioscience 66, 938–948. 10.1093/biosci/biw111 28533562 PMC5421442

[B42] Inglis G. J. (2000). Disturbance-related heterogeneity in the seed banks of a marine angiosperm. J. Ecol. 88, 88–99. 10.1046/j.1365-2745.2000.00433.x

[B43] Jackson J. B. Kirby M. X. Berger W. H. Bjorndal K. A. Botsford L. W. Bourque B. J.. (2001). Historical overfishing and the recent collapse of coastal ecosystems. Science 293, 629–638. 10.1126/science.1059199 11474098

[B44] Jannsen T. Bremer K. (2004). The age of major monocot groups inferred from 800+ rbcL sequences. Bot. J. Linn. Soc. 146, 385–398. 10.1111/j.1095-8339.2004.00345.x

[B45] Kamvar Z. N. Tabima J. F. Gruenwald N. J. (2014). Poppr: an R package for genetic analysis of populations with clonal, partially clonal, and/or sexual reproduction. PeerJ 2:e281 10.7717/peerj.281 24688859 PMC3961149

[B46] Kendrick G. A. Orth R. J. Statton J. Hovey R. Ruiz Montoya L. R. Lowe R. J.. (2017). Demographic and genetic connectivity: the role and consequences of reproduction, dispersal and recruitment in seagrasses. Biol. Rev. 92, 921–938. 10.1111/brv.12261 27010433

[B47] Kendrick G. A. Waycott M. Carruthers T. J. B. Cambridge M. L. Hovey R. Krauss S. L. (2012). The central role of dispersal in the maintenance and persistence of seagrass populations. Bioscience 62, 56–65. 10.1525/bio.2012.62.1.10

[B48] Kilminster K. McMahon K. Waycott M. Kendrick G. A. Scanes P. McKenzie L.. (2015). Unravelling complexity in seagrass systems for management: Australia as a microcosm. Sci. Tot. Environ. 534, 97–109. 10.1016/j.scitotenv.2015.04.061 25917445

[B49] Klotzbach P. J. (2011). El Ni-o–Southern oscillation's impact on Atlantic Basin hurricanes and U.S. landfalls. J. Clim. 24, 1252–1263. 10.1175/2010JCLI3799.1

[B50] Lee K. S. Park S. R. Kim Y. K. (2007). Effects of irradiance, temperature, and nutrients on growth dynamics of seagrasses: a review. J. Exp. Mar. Biol. Ecol. 350, 144–175. 10.1016/j.jembe.2007.06.016

[B51] Les D. H. Cleland M. A. Waycott M. (1997). Phylogenetic studies in Alismatidae, II: evolution of marine angiosperms (seagrasses) and hydrophily. Syst. Bot. 22, 443–463. 10.2307/2419820

[B52] Lough J. M. (1998). Coastal climate of northwest Australia and comparisons with the Great Barrier Reef: 1960 to 1992. Coral Reefs 17, 351–367. 10.1007/s003380050139

[B53] Marsh H. Sobtzick S. (2015). Dugong dugon. The IUCN Red List of Threatened Species 2015. e.T6909A43792211. 10.2305/IUCN.UK.2015-4.RLTS.T6909A43792211.en

[B54] Marsh H. O'Shea T. J. Reynolds J. E. (2012). Ecology and Conservation of the Sirenia: Dugongs and Manatees. Cambridge: Cambridge University Press.

[B55] McConochie J. D. Hardy T. A. Mason L. B. (2004). Modelling tropical cyclone over-water wind and pressure fields. Ocean Eng. 31, 1757–1782. 10.1016/j.oceaneng.2004.03.009

[B56] McMahon K. van Dijk K.-j. Ruiz-Montoya L. Kendrick G. A. Krauss S. L. Waycott M.. (2014). The movement ecology of seagrasses. Proc. R. Soc. B Biol. Sci. 281:20140878. 10.1098/rspb.2014.0878 25297859 PMC4213608

[B57] Myers N. Mittermeier R. A. Mittermeier C. G. da Fonseca G. A. Kent J. (2000). Biodiversity hotspots for conservation priorities. Nature 403, 853–858. 10.1038/35002501 10706275

[B58] Olesen B. Marba N. Duarte C. M. Savela R. S. Fortes M. D. (2004). Recolonization dynamics in a mixed seagrass meadow: the role of clonal versus sexual processes. Estuaries 27, 770–780. 10.1007/BF02912039

[B59] Olsen J. L. Stam W. T. Coyer J. A. Reusch T. B. H. Billingham M. Bostr Ö. M. C.. (2004). North Atlantic phylogeography and large-scale population differentiation of the seagrass *Zostera marina* L. Mol. Ecol. 13, 1923–1941. 10.1111/j.1365-294X.2004.02205.x 15189214

[B60] Ooi J. L. S. Kimberly P. Gary A. Karen W. (2014). Spatial structure of seagrass suggests that size-dependent plant traits have a strong influence on the distribution and maintenance of tropical multispecies meadows. PLoS ONE 19:e86782 10.1371/journal.pone.0086782 PMC3909009 24497978

[B61] Peakall R. O. D. Smouse P. E. (2006). genalex 6: genetic analysis in Excel. Population genetic software for teaching and research. Mol. Ecol. Notes 6, 288–295. 10.1111/j.1471-8286.2005.01155.x PMC3463245 22820204

[B62] Peterson B. K. Weber J. N. Kay E. H. Fisher H. S. Hoekstra H. E. (2012). Double Digest RADseq: an inexpensive method for *de novo* SNP discovery and genotyping in model and non-model species. PLoS ONE 7:e37135. 10.1371/journal.pone.0037135 22675423 PMC3365034

[B63] Pope L. C. Riginos C. Ovenden J. Keyse J. Blomberg S. P. (2015). Population genetic diversity in the Australian 'Seascape': a bioregion approach. PLoS ONE 10:e0136275. 10.1371/journal.pone.0136275 26375711 PMC4574161

[B64] Preen A. (1995). Impacts of dugong foraging on seagrass habitats - observational and experimental evidence for cultivation grazing. Mar. Ecol. Prog. Ser. 124, 201–213. 10.3354/meps124201

[B65] Puotinen M. Maynard J. A. Beeden R. Radford B. Williams G. J. (2016). A robust operational model for predicting where tropical cyclone waves damage coral reefs. Sci. Rep. 6:26009. 10.1038/srep26009 27184607 PMC4868967

[B66] Quiros T. E Croll D. Tershy B. Fortes M. D. Raimondi P. (2017). Land use is a better predictor of tropical seagrass condition than marine protection. Biol. Conserv. 209, 454–463. 10.1016/j.biocon.2017.03.011

[B67] Ralph P. J. Durako M. J. Enriquez S. Collier C. J. Doblin M. A. (2007). Impact of light limitation on seagrasses. J. Exp. Mar. Biol. Ecol. 350, 176–193. 10.1016/j.jembe.2007.06.017

[B68] Rasheed M. A. (2004). Recovery and succession in a multi-species tropical seagrass meadow following experimental disturbance: the role of sexual and asexual reproduction. J. Exp. Mar. Biol. Ecol. 310, 13–45. 10.1016/j.jembe.2004.03.022

[B69] Reisch C. Scheitler S. (2009). Disturbance by mowing affects clonal diversity: the genetic structure of *Ranunculus ficaria* (Ranunculuaceae) in meadows and forests, in Herbaceous Plant Ecology: Recent Advances in Plant Ecology, ed A. G. Van der Valk (Dordrecht: Springer), 335–343. 10.1007/978-90-481-2798-6_28

[B70] Renema W. Bellwood D. R. Braga J. C. Bromfield K. Hall R. Johnson K. G.. (2008). Hopping hotspots: global shifts in marine biodiversity. Science 321, 654–657. 10.1126/science.1155674 18669854

[B71] Reusch T. B. H. (2006). Does disturbance enhance genotypic diversity in clonal organisms? A field test in the marine angiosperm *Zostera marina*. Mol. Ecol. 15, 277–286. 10.1111/j.1365-294X.2005.02779.x 16367846

[B72] Reusch T. B. H. Ehlers A. Hämmerli A. Worm B. (2005). Ecosystem recovery after climatic extremes enhanced by genotypic diversity. Proc. Natl. Acad. Sci. U.S.A. 102, 2826–2831. 10.1073/pnas.0500008102 15710890 PMC549506

[B73] Reusch T. B. H. Stam W. T. Olsen J. L. (1999). Size and estimated age of genets in eelgrass, *Zostera marina*, assessed with microsatellite markers. Mar. Biol. 133, 519–525. 10.1007/s002270050492

[B74] Salo T. Reusch T. B. H. Bostrom C. (2015). Genotype-specific responses to light stress in eelgrass *Zostera marina*, a marine foundation plant. Mar. Ecol. Prog. Ser. 519, 129–140. 10.3354/meps11083

[B75] Sebens K. P. Thorne B. I. (1985). Coexistence of clones, clonal diversity, and the effects of disturbance, in Population Biology and Evolution of Clonal Organisms, eds Jackson J. B. C. Buss L. W. Cook R. E. (New Haven, CT: Yale University Press), 357–398.

[B76] Short F. Carruthers T. Dennison W. Waycott M. (2007). Global seagrass distribution and diversity: a bioregional model. J. Exp. Mar. Biol. Ecol. 350, 3–20. 10.1016/j.jembe.2007.06.012

[B77] Short F. T. Polidoro B. Livingstone S. R. Carpenter K. E. Bandeira S. Bujang J. S. (2011). Extinction risk assessment of the world's seagrass species. Biol. Conserv. 144, 1961–1971. 10.1016/j.biocon.2011.04.010

[B78] Sinclair E. A. Krauss S. L. Anthony J. Hovey R. Kendrick G. A. (2014). The interaction of environment and genetic diversity within meadows of the seagrass *Posidonia australis* (Posidoniaceae). Mar. Ecol. Prog. Ser. 506, 87–98. 10.3354/meps10812

[B79] Sobel A. H. Camargo S. J. Hall T. M. Lee C.-Y. Tippett M. K. Wing A. A. (2016). Human influence on tropical cyclone intensity. Science 353, 242–246. 10.1126/science.aaf6574 27418502

[B80] Stenström A. Jonsson B. O. Jónsdóttir I. S. Fagerström T. Augner M. (2001). Genetic variation and clonal diversity in four clonal sedges (Carex) along the Arctic coast of Eurasia. Mol. Ecol. 10, 497–513. 10.1046/j.1365-294x.2001.01238.x 11298963

[B81] Tartaglione C. A. Smith S. R. O'Brien J. J. (2003). ENSO impact on hurricane landfall probabilities for the Caribbean. J. Clim. 16, 2925–2931. 10.1175/1520-0442(2003)016<2925:EIOHLP>2.0.CO;2

[B82] Thomas L. Kendrick G. Stat M. Travaille K. Shedrawi G. Kennington W. (2014). Population genetic structure of the *Pocillopora damicornis* morphospecies along Ningaloo Reef, Western Australia. Mar. Ecol. Prog. Ser. 513, 111–119. 10.3354/meps10893

[B83] Thomas L. Kennington W. J. Evans R. D. Kendrick G. A. Stat M. (2017). Restricted gene flow and local adaptation highlight the vulnerability of high-latitude reefs to rapid environmental change. Glob. Chang. Biol. 23, 1365–2486. 10.1111/gcb.13639 28132420

[B84] Thomson J. A. Burkholder D. A. Heithaus M. R. Fourqurean J. W. Fraser M. W. Statton J.. (2015). Extreme temperatures, foundation species, and abrupt ecosystem change: an example from an iconic seagrass ecosystem. Glob. Chang. Biol. 21, 1463–1474. 10.1111/gcb.12694 25145694

[B85] Tittensor D. P. Mora C. Jetz W. Lotze H. K. Ricard D. Berghe E. V.. (2010). Global patterns and predictors of marine biodiversity across taxa. Nature 466, 1098–1101. 10.1038/nature09329 20668450

[B86] Tol S. J. Jarvis J. C. York P. H. Grech A. Congdon B. C. Coles R. G. (2017). Long distance biotic dispersal of tropical seagrass seeds by marine mega-herbivores. Sci. Rep. 7, 4458. 10.1038/s41598-017-04421-1 28667257 PMC5493642

[B87] Tolman H. L. (2009). User Manual and System Documentation of WAVEWATCH III TM version 3.14. U. S. Department of Commerce, National Oceanic and Atmospheric Administration, National Weather Service, National Centers for Environmental Prediction, Camp Springs, MD.

[B88] Unsworth R. K. Collier C. J. Waycott M. McKenzie L. J. Cullen-Unsworth L. C. (2015). A framework for the resilience of seagrass ecosystems. Mar. Pollut. Bull. 100, 34–46. 10.1016/j.marpolbul.2015.08.016 26342389

[B89] van Dijk K.-J. Mellors J. Waycott M. (2014). Development of multiplex microsatellite PCR panels for the seagrass *Thalassia hemprichii* (Hydrocharitaceae). Appl. Plant Sci. 2:1400078. 10.3732/apps.1400078 25383269 PMC4222546

[B90] van Dijk J. K. van Tussenbroek B. I. (2010). Clonal diversity and structure related to habitat of the marine angiosperm *Thalassia testudinum* along the Atlantic coast of Mexico. Aquat. Bot. 92, 63–69. 10.1016/j.aquabot.2009.10.005

[B91] van Groenendael J. M. Klimes L. Klimesova J. Hendriks R. J. J. (1996). Plant life histories: ecological correlates and phylogenetic constraints - comparative ecology of clonal plants. Philos. Transact. R. Soc. Lond. Ser. B 351, 1331–1339. 10.1098/rstb.1996.0116

[B92] van Tussenbroek B. I. Valdivia-Carrillo T. Rodríguez-Virgen I. T. Sanabria-Alcaraz S. N. M. Jiménez-Durán K. van Dijk K.-j.. (2016). Coping with potential bi-parental inbreeding: limited pollen and seed dispersal and large genets in the dioecious marine angiosperm *Thalassia testudinum*. Ecol. Evol. 6, 5542–5556. 10.1002/ece3.2309 27942375 PMC5127610

[B93] van Tussenbroek B. I. Vonk J. A. Stapel J. Erftemeijer P. L. A. Middelburg J. J. Zieman J. C. (2006). The biology of Thalassia: paradigms and recent advances in research, in Seagrasses: Biology, Ecology and Conservation, eds Larkum A. W. D. Orth R. J. Duarte C. M. (Amsterdam: Elsevier), 409–439.

[B94] Vellend M. Lajoie G. Bourret A. Múrria C. Kembel S. W. Garant D. (2014). Drawing ecological inferences from coincident patterns of population- and community-level biodiversity. Mol. Ecol. 23, 2890–2901. 10.1111/mec.12756 24750409

[B95] Vik U. Jørgensen M. H. Kauserud H. Nordal I. Brysting A. K. (2010). Microsatellite markers show decreasing diversity but unchanged level of clonality in *Dryas octopetala* (Rosaceae) with increasing latitude. Am. J. Bot. 97, 988–997. 10.3732/ajb.0900215 21622468

[B96] Villacorta-Rath C. Ilyushkina I. Strugnell J. M. Green B. S. Murphy N. P. Doyle S. R. (2016). Outlier SNPs enable food traceability of the southern rock lobster, *Jasus edwardsii*. Mar. Biol. 163, 223 10.1007/s00227-016-3000-1

[B97] Wainwright B. J. Arlyza I. S. Karl S. A. (2013). Isolation and characterization of twenty-four polymorphic microsatellite loci for the tropical seagrass, *Thalassia hemprichii*. Conserv. Genet. Resour. 5, 939–941. 10.1007/s12686-013-9937-1

[B98] Waits L. P. Luikart G. Taberlet P. (2001). Estimating the probability of identity among genotypes in natural populations: cautions and guidelines. Mol. Ecol. 10, 249–256. 10.1046/j.1365-294X.2001.01185.x 11251803

[B99] Wang M. Son S. Harding L. (2009). Retrieval of diffuse attenuation coefficient in the Chesapeake Bay and turbid ocean regions for satellite ocean color applications. J. Geophys. Res. 114, C10 10.1029/2009JC005286

[B100] Waycott M. Collier C. J. McMahon K. Ralph P. J. McKenzie L. J. Udy J. (2007). Vulnerability of seagrasses in the Great Barrier Reef to climate change, in Climate Change and the Great Barrier Reef: A Vulnerability Assessment, eds Johnson J. E. Marshall P. (Townsville, QLD: Great Barrier Reef Marine Park Authority and Australian Greenhouse Office), 193–235.

[B101] Waycott M. McMahon K. M. Lavery P. S. (2014). A Guide to Temperate Southern Seagrasses. Melbourne, VIC: CSIRO.

[B102] Waycott M. McMahon K. M. Mellors J. E. Calladine A. Kleine D. (2004). A Guide to Tropical Seagrasses of the Indo-West Pacific. James Cook University, Townsville, QLD.

[B103] Williams S. L. (1988). Disturbance and recovery of a deep-water Caribbean seagrass bed. Mar. Ecol. Prog. Ser. 42, 63–71. 10.3354/meps042063

[B104] Wood S. N. Scheipl F. (2015). gamms: Generalized Additive Mixed Models using mgcv and lme4. R package. Available online at: https://cran.r-project.org/web/packages/gamm4/index.html

[B105] Wu K. Chen C.-N. Soong K. (2016). Long distance dispersal potential of two seagrasses *Thalassia hemprichii* and *Halophila ovalis*. PLoS ONE 11:e0156585. 10.1371/journal.pone.0156585 27248695 PMC4889049

[B106] Xu N. N. Yu S. Zhang J. G. Tsang P. K. E. Chen X. Y. (2010). Microsatellite primers for *Halophila ovalis* and cross-amplification in *H. minor* (Hydrocharitaceae). Am. J. Bot. 97, E56–E57. 10.3732/ajb.1000111 21622460

[B107] Ye D. Hu Y. Song M. Pan X. Xie X. Liu G.. (2014). Clonality-climate relationships along latitudinal gradient across China: adaptation of clonality to environments. PLoS ONE 9:e94009. 10.1371/journal.pone.0094009 24709992 PMC3977992

[B108] Ye D. Liu G. Song Y.-B. Cornwell W. K. Dong M. Cornelissen J. H. C. (2016). Strong but diverging clonality - climate relationships of different plant clades explain weak overall pattern across China. Sci. Rep. 6:26850. 10.1038/srep26850 27246203 PMC4887789

[B109] Zinke J. Gilmour J. Fisher R. Puotinen M. Maina J. Darling E. (in press). Gradients of disturbance, environmental conditions and coral community structure for south-eastern Indian Ocean reefs. Divers. Distribut.

[B110] Zipperle A. M. Coyer J. A. Reise K. Stam W. T. Olsen J. L. (2010). Waterfowl grazing in autumn enhances spring seedling recruitment of intertidal *Zostera noltii*. Aquat. Bot. 93, 202–205. 10.1016/j.aquabot.2010.05.002

