# Supplementary material for: Disturbance Is an Important Driver of Clonal Richness in Tropical Seagrasses
Source: Front Plant Sci. 2017 Dec 5;8:2026. doi: 10.3389/fpls.2017.02026 (PMC5723400; doi:10.3389/fpls.2017.02026)
Supplement: Supplementary file 1 [file Table1.docx]

Supplementary Material

Disturbance is an important driver of clonal richness in tropical seagrasses

Kathryn McMahon^*^, Richard D. Evans, Kor-jent van Dijk, Udhi Hernawan, Gary Kendrick, Paul S. Lavery, Ryan Lowe, Marji Puotinen and Michelle Waycott

*** Correspondence:** Corresponding Author: k.mcmahon@ecu.edu.au

**Supplementary Table 1: A list of all sites sampled and the clonal richness estimates. N=number of samples genotyped, G=number of MLG’s detected, R=clonal richness, P_ID_= Probability of identify, Est MLG=estimated number of MLG’s based on the P_ID_ and N. The remaining columns tabulate the environmental drivers used in the GAMMs analysis but these were not collated for *Thalassia hemprichii*.**

***Thalassia hemprichii***

| **ID** | **Site** | **Latitude** | **Longitude** | **N** | **G** | **R** | **P_ID_** | **Est MLG** | **Dist.**  **Shore** | **Dugong** | **KD490** | **SST** | **Cyclone** |
| --- | --- | --- | --- | --- | --- | --- | --- | --- | --- | --- | --- | --- | --- |
| 1 | Biak | -1.09916 | 136.35775 | 48 | 48 | 1.00 | <0.001 | <0.001 |  |  |  |  |  |
| 2 | Tual | -5.52244 | 132.79834 | 48 | 47 | 0.98 | <0.001 | <0.001 |  |  |  |  |  |
| 3 | Ambon | -3.65379 | 128.19972 | 48 | 24 | 0.49 | <0.001 | <0.001 |  |  |  |  |  |
| 4 | Kendari | -3.65379 | 122.60996 | 48 | 38 | 0.79 | <0.001 | <0.001 |  |  |  |  |  |
| 5 | Bitung | 1.38896 | 125.10199 | 48 | 48 | 1.00 | <0.001 | <0.001 |  |  |  |  |  |
| 6 | Palu | -0.74837 | 119.85702 | 48 | 39 | 0.81 | <0.001 | <0.001 |  |  |  |  |  |
| 7 | Jepara | -6.57563 | 110.63142 | 48 | 29 | 0.60 | <0.001 | <0.001 |  |  |  |  |  |
| 8 | Pari Is. | -5.86387 | 106.61068 | 48 | 36 | 0.74 | <0.001 | <0.001 |  |  |  |  |  |
| 9 | Bangka^++^ | -2.97466 | 106.65222 | 48 | 5 | 0.09 | 0.001 | 0.037 |  |  |  |  |  |
| 10 | Natuna | 3.95256 | 108.3993 | 48 | 37 | 0.77 | <0.001 | <0.001 |  |  |  |  |  |
| 11 | Kupang | -10.13309 | 123.65807 | 48 | 43 | 0.89 | <0.001 | <0.001 |  |  |  |  |  |
| 12 | Lombok | -8.40368 | 116.07957 | 48 | 44 | 0.91 | <0.001 | <0.001 |  |  |  |  |  |
| 13 | Drini | -8.13870 | 110.57870 | 48 | 36 | 0.74 | <0.001 | 0.014 |  |  |  |  |  |
| 14 | Padang | -1.01637 | 100.38954 | 48 | 40 | 0.83 | <0.001 | <0.001 |  |  |  |  |  |
| 15 | Cocos Keeling | -12.19832 | 96.84287 | 48 | 43 | 0.89 | 0.001 | 0.041 |  |  |  |  |  |
| 16 | Kimberley | -16.432222 | 123.342706 | 48 | 44 | 0.91 | 0.002 | 0.075 |  |  |  |  |  |
| 17 | Exmouth | -21.64733 | 114.37423 | 48 | 39 | 0.81 | 0.001 | 0.048 |  |  |  |  |  |

***Halodule uninervis***

| **ID** | **Site** | **Latitude** | **Longitude** | **N** | **G** | **R** | **P_ID_** | **Est MLG** | **Dist.**  **shore** | **Dugong** | **KD490** | **SST** | **Cyclone** |
| --- | --- | --- | --- | --- | --- | --- | --- | --- | --- | --- | --- | --- | --- |
| 1 | Broome 1 | 122.335986 | -17.983188 | 39 | 22 | 0.55 | <0.001 | <0.001 | 0.70 | 1 | 0.140 | 27.9 | 0.000 |
| 2 | Broome 2 | 122.242035 | -17.960715 | 35 | 28 | 0.79 | <0.001 | <0.001 | 0.90 | 1 | 0.140 | 27.9 | 0.032 |
| 3 | Balla Balla 1 | 117.686233 | -20.64498 | 45 | 32 | 0.70 | <0.001 | <0.001 | 3.50 | 1 | 0.169 | 27.5 | 0.097 |
| 4 | Balla Balla 2 | 117.709178 | -20.650356 | 42 | 31 | 0.73 | <0.001 | <0.001 | 2.50 | 1 | 0.169 | 27.5 | 0.065 |
| 5 | Rosemary Is. 1 | 116.610594 | -20.45776 | 47 | 3 | 0.04 | <0.001 | <0.001 | 21.00 | 0 | 0.066 | 26.9 | 0.355 |
| 6 | Rosemary Is. 2 | 116.609229 | -20.4618 | 34 | 3 | 0.06 | <0.001 | <0.001 | 20.50 | 0 | 0.066 | 26.9 | 0.355 |
| 7 | Montebello Is. 1 | 115.5059252 | -20.38328941 | 42 | 1 | 0.00 | <0.001 | <0.001 | 88.00 | 0 | 0.085 | 26.3 | 0.387 |
| 8 | Montebello Is. 2 | 115.5074165 | -20.43323885 | 47 | 6 | 0.11 | <0.001 | <0.001 | 83.00 | 0 | 0.083 | 26.6 | 0.387 |
| 9 | Thevenard Is. 1 | 114.9885375 | -21.4495 | 48 | 8 | 0.15 | <0.001 | <0.001 | 20.50 | 1 | 0.112 | 26.5 | 0.290 |
| 10 | Thevenard Is. 2 | 114.9909245 | -21.44884 | 48 | 5 | 0.09 | <0.001 | <0.001 | 23.00 | 1 | 0.166 | 26.5 | 0.258 |
| 11 | Exmouth Gulf 1 | 114.35373 | -22.31613 | 31 | 23 | 0.73 | <0.001 | <0.001 | 13.50 | 1 | 0.231 | 26.0 | 0.097 |
| 12 | Exmouth Gulf 2 | 114.35344 | -22.31444 | 42 | 27 | 0.63 | <0.001 | <0.001 | 14.50 | 1 | 0.231 | 26.0 | 0.065 |
| 13 | Ningaloo 1 | 113.91946 | -21.9798 | 46 | 1 | 0.00 | <0.001 | <0.001 | 1.50 | 0 | 0.058 | 25.8 | 0.194 |
| 14 | Lake Macleod | 113.6466667 | -23.9519444 | 7 | 1 | 0.00 | <0.001 | <0.001 | na | na | na | na | na |
| 15 | Shark Bay 1 | 113.5764995 | -26.0942045 | 36 | 9 | 0.23 | <0.001 | <0.001 | 0.34 | 1 | 0.190 | 23.6 | 0.000 |
| 16 | Shark Bay 2 | 113.55378 | -26.0305756 | 44 | 15 | 0.33 | <0.001 | <0.001 | 1.51 | 1 | 0.283 | 23.9 | 0.000 |

***Halophila ovalis***

| **ID** | **Site** | **Latitude** | **Longitude** | **N** | **G** | **R** | **P_ID_** | **Est MLG** | **Dist.**  **shore** | **Dugong** | **KD490** | **SST** | **Cyclone** |
| --- | --- | --- | --- | --- | --- | --- | --- | --- | --- | --- | --- | --- | --- |
| 1 | Broome 2 | 122.242035 | -17.960715 | 33 | 32 | 0.97 | <0.001 | 0.017 | 0.90 | 1 | 0.140 | 27.9 | 0.032 |
| 2 | Rosemary Is. 1. | 116.610594 | -20.45776 | 46 | 9 | 0.18 | 0.003 | 0.100 | 21.00 | 0 | 0.066 | 26.9 | 0.355 |
| 3 | Rosemary Is. 2. | 116.609229 | -20.4618 | 47 | 5 | 0.09 | 0.004 | 0.200 | 20.50 | 0 | 0.066 | 26.9 | 0.355 |
| 4 | Montebello Is. 2 | 115.5074165 | -20.4332388 | 33 | 2 | 0.03 | 0.021 | 0.700 | 83.00 | 0 | 0.083 | 26.3 | 0.387 |
| 5 | Thevenard Is. 1. | 114.9885375 | -21.4495 | 13 | 4 | 0.25 | 0.032 | 0.400 | 20.50 | 1 | 0.112 | 26.5 | 0.290 |
| 6 | Thevenard Is. 2. | 115.0165 | -21.4654 | 42 | 19 | 0.44 | 0.001 | 0.100 | 23.00 | 1 | 0.166 | 26.5 | 0.258 |
| 7 | Muiron Is. North | 114.37423 | -21.64733 | 33 | 9 | 0.25 | 0.001 | 0.024 | 26.00 | 0 | 0.105 | 26.0 | 0.226 |
| 8 | Muiron Is. South | 114.33107 | -21.68615 | 46 | 12 | 0.24 | <0.001 | 0.005 | 20.50 | 0 | 0.129 | 26.0 | 0.226 |
| 9 | Exmouth Gulf 1 | 114.35373 | -22.31613 | 45 | 42 | 0.93 | <0.001 | 0.002 | 13.50 | 1 | 0.231 | 26.0 | 0.097 |
| 10 | Exmouth Gulf 2 | 114.35344 | -22.31444 | 49 | 47 | 0.96 | 0.026 | 0.003 | 14.50 | 1 | 0.231 | 26.0 | 0.065 |
| 11 | Ningaloo 1 | 113.91946 | -21.9798 | 41 | 12 | 0.28 | <0.001 | 0.017 | 1.50 | 0 | 0.058 | 25.8 | 0.194 |
| 12 | Ningaloo 2 | 113.9197496 | -22.0079061 | 47 | 8 | 0.15 | 0.016 | 0.750 | 0.70 | 0 | 0.065 | 25.7 | 0.194 |
| 13 | Shark Bay 1 | 113.5764995 | -26.0942045 | 47 | 6 | 0.11 | 0.014 | 0.700 | 0.34 | 1 | 0.190 | 23.6 | 0.000 |
| 14 | Shark Bay 2. | 113.55378 | -26.0305756 | 47 | 16 | 0.33 | 0.026 | 1.200 | 1.51 | 1 | 0.283 | 23.9 | 0.000 |
