# Supplementary material for: Disturbance Is an Important Driver of Clonal Richness in Tropical Seagrasses
Source: Front Plant Sci. 2017 Dec 5;8:2026. doi: 10.3389/fpls.2017.02026 (PMC5723400; doi:10.3389/fpls.2017.02026)
Supplement: Supplementary file 2 [file Table2.docx]

Supplementary Material

Disturbance is an important driver of clonal richness in tropical seagrasses

Kathryn McMahon^*^, Richard D. Evans, Kor-jent van Dijk, Udhi Hernawan, Gary Kendrick, Paul S. Lavery, Ryan Lowe, Marji Puotinen and Michelle Waycott

*** Correspondence:** Corresponding Author: k.mcmahon@ecu.edu.au

**Supplementary Table 2**: Correlation coefficients between species and latitude and the two species, *H. uninervis* and *H.ovalis* where both species co-occurred. r=correlation coefficient, p=probability, n=number of sites in analysis.

| Species | Latitude | *H. uninervis* |
| --- | --- | --- |
| *H. uninervis* | r=-0.255  p=0.340  n=16 |  |
| *H.ovalis* | r=-0.213  p=0.465  n=14 | r=0.806  p=0.003  n=11 |
| *T.hemprichii* | r=0.162  p=0.534  n=17 |  |
