# Supplementary material for: Disturbance Is an Important Driver of Clonal Richness in Tropical Seagrasses
Source: Front Plant Sci. 2017 Dec 5;8:2026. doi: 10.3389/fpls.2017.02026 (PMC5723400; doi:10.3389/fpls.2017.02026)
Supplement: Supplementary file 3 [file Table3.docx]

Supplementary Material

Disturbance is an important driver of clonal richness in tropical seagrasses

Kathryn McMahon^*^, Richard D. Evans, Kor-jent van Dijk, Udhi Hernawan, Gary Kendrick, Paul S. Lavery, Ryan Lowe, Marji Puotinen and Michelle Waycott

*** Correspondence:** Corresponding Author: k.mcmahon@ecu.edu.au


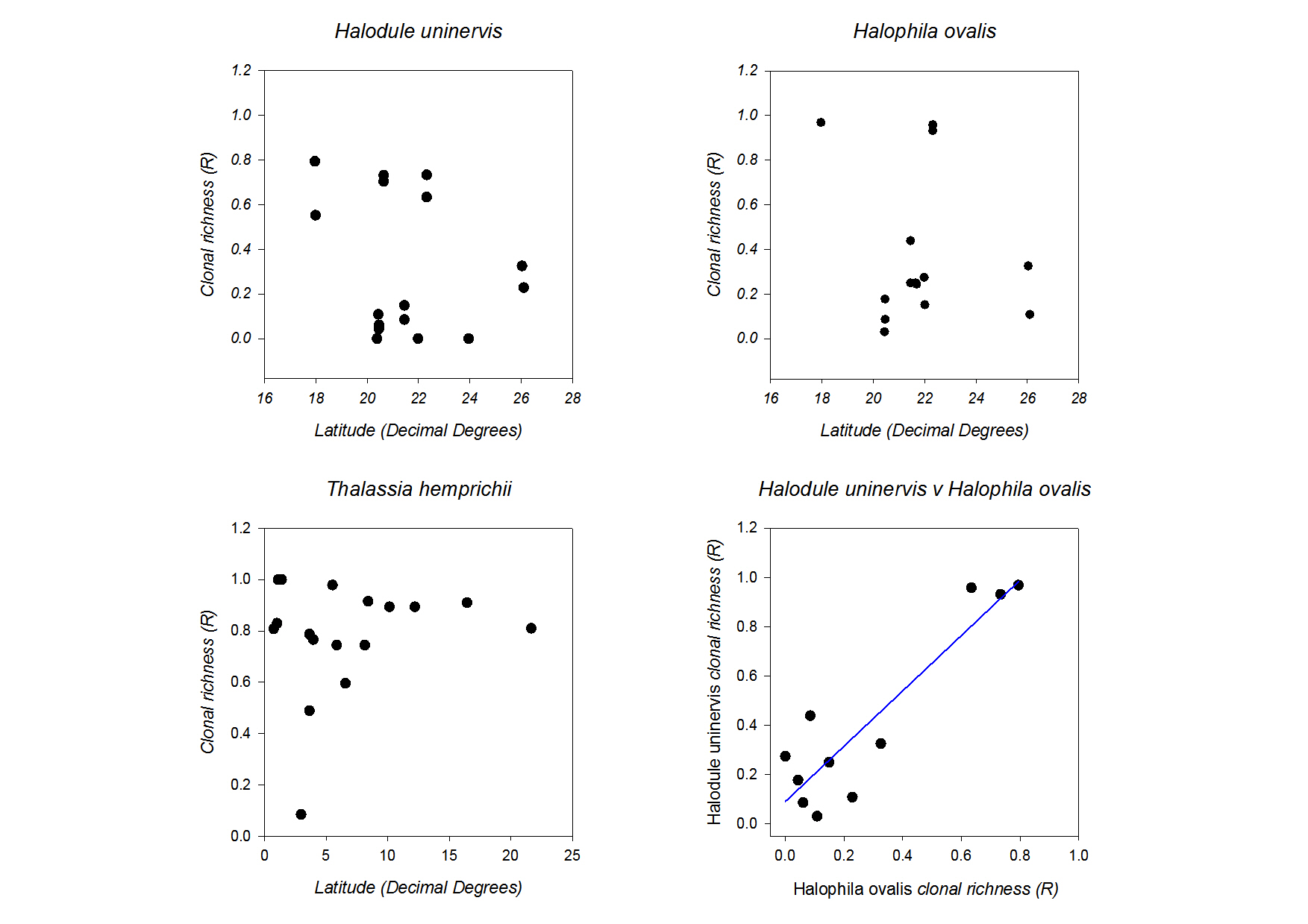


**Supplementary Figure 1**: There were no significant correlations of clonal richness with latitude for *H.* uninervis, *H. ovalis* and *T. hemprichii* but where *H*. uninervis and *H. ovalis* co-occurred there was a significant positive correlation with clonal richness. Based on Pearson correlation coefficient. See Supplementary Table 2 for statistical outputs.
